# Inaccurate and misleading meta-analysis of E-cigarettes and population-based diseases

**DOI:** 10.1007/s11739-025-03956-w

**Published:** 2025-05-12

**Authors:** Brad Rodu, Nantaporn Plurphanswat, Jordan Rodu

**Affiliations:** 1 James Graham Brown Cancer Center, University of Louisville, Louisville, KY USA; 2 Department of Medicine, School of Medicine, University of Louisville, 505 South Hancock Street, Louisville, KY 40202 USA; 3 Department of Statistics, University of Virginia, Charlottesville, VA USA

**Keywords:** Meta-analysis, E-cigarettes, Cardiovascular disease, Stroke, Chronic obstructive pulmonary disease, Cross-sectional surveys, Longitudinal surveys

## Abstract

A random-effects meta-analysis by Glantz et al. recently concluded that the odds of several diseases among current e-cigarette users and smokers were similar. This report details serious deficiencies. We used descriptive analysis methods to assess the studies the authors selected for cardiovascular disease (CVD), stroke and chronic obstructive pulmonary disease (COPD) among e-cigarette users vs. nonusers. We examined all of the source studies for these categories. We demonstrate that the meta-analysis by Glantz et al. had three principal deficits that were avoidable: (1) mixing unjustified and incomprehensible disease outcomes, such as erectile dysfunction with fatal CVDs and influenza with COPD; (2) using survey datasets containing no temporal information about smoking/vaping initiation and disease diagnosis; (3) using longitudinal studies that didn’t account for changes in vaping and smoking during follow-up waves. The meta-analysis by Glantz et al. is misleading and inaccurate. The deficits are only apparent to investigators thoroughly experienced with the data from the source studies. We conclude that Glantz et al. failed to meet basic criteria for the quality of source studies; the results of their meta-analysis are invalid.

## Introduction

Since electronic cigarettes (e-cigarettes) entered the U.S. market in 2007 [1], the health consequences have been a subject of controversy among public health researchers. Despite the fact that e-cigarettes do not produce as many toxic chemicals as combustible cigarettes [2], many researchers have used observational studies to claim that e-cigarette use increases the risk of smoking-related diseases, especially cardiovascular and respiratory diseases.

Glantz et al. [3] recently published a random-effects meta-analysis entitled “population-based disease odds for e-cigarettes and dual use versus cigarettes.” The authors “identified 124 odds ratios (94 cross-sectional and 30 longitudinal) from 107 studies,” and they concluded that “Direct epidemiological evidence based on actual use of e-cigarettes in the general population suggests that, at least for cardiovascular disease, stroke, and metabolic dysfunction, the odds of disease between current e-cigarette and cigarette use were similar.”

At first glance, the meta-analyses appear to be robust and convincing, because their pooled odds ratios (ORs) are often statistically significant. However, after publication, several issues were raised in letters on the journal website [4, 5]. These include the small number and short exposure durations of e-cigarette users in the source studies, lack of dose–response assessment [4], and two serious methodologic flaws involving assessing bias and certainty of evidence [5].

After a careful review, we document that Glantz et al. inexplicably commingled outcomes in cardiovascular disease (CVD) and chronic obstructive pulmonary disease (COPD) categories. Next, we demonstrate that the quality of source studies selected by Glantz et al. failed to meet a basic criterion, described by Egger et al. [6] as “Garbage in—garbage out?: The quality of component trials is of crucial importance: if the ‘raw material’ is flawed, the findings of reviews of this material may also be compromised. Clearly, the trials included in systematic reviews and meta-analyses should ideally be of high methodological quality…” Specifically, we show that (a) cross-sectional studies have been proven entirely deficient in crucial temporal information about exposure and disease diagnosis and, (b) existing longitudinal studies from the FDA’s Population Assessment of Tobacco and Health (PATH) survey are inadequately adjusted for changes in exposure during follow-up. Finally, we discuss other important analytic errors.

We will show that Glantz et al., failed to meet these quality control standards, which is crucial in meta-analyses [6, 7]. Consequently, their findings are inaccurate and unreliable.

## Methods

In order to provide clear examples of the low-quality raw materials used by Glantz et al., we assessed the studies they selected for CVD, stroke and chronic obstructive pulmonary disease (COPD) among e-cigarette users vs. nonusers, as listed in Figure S2 and Table S2 of their Appendix [3]. We examined all of the source studies for these categories, particularly focusing on specific disease outcomes, data source(s), and relative risks (RRs) or odds ratios (ORs). Our results are displayed as descriptive tables.

## Results

### Mixed disease outcomes

The characteristics of the studies analyzed in the three disease outcome categories [8–37] are seen in Tables 1 and 2. First, there are considerable irregularities in how Glantz et al. classified disease outcomes and included them in calculating pooled ORs.

**Table 1:** Characteristics of cross-sectional studies used in meta-analysis of cardiovascular disease, stroke and COPD among e-cigarette users Vs. nonusers. Glantz et al. [3], Figure S2, Supplement

Glantz Disease Category Detailed study outcome(s)	Study First Author (Reference), year	Data source	Year/Wave(s)	RR/OR (CI)
Cardiovascular disease				
Erectile dysfunction	El-Shahawy [8], 2022	PATH	W4	2.24 (1.50–3.34)
Congestive HF	Gathright [9], 2019		W1	1.49 (0.77–2.88)
MI	Alzahrani [10], 2018	NHIS	2014, 2016	1.79 (1.20–2.67)
	Falk [11], 2022		2014, 2016–18	0.98 (0.55–1.73)
Coronary heart disease	Farsalinos [12], 2019	NHIS	2016–17	1.31 (0.79–2.17)
Coronary heart disease, MI, stroke	Liu [13], 2022	BRFSS	2020	1.17 (0.97–1.41)
	Osei [14], 2019		2016–17	1.04 (0.63–1.72)
Stroke				
Stroke	Bricknell [19], 2021	BRFSS	2016	1.62 (1.16–2.27)
	Falk [11], 2022		2014, 2016–18	1.06 (0.71–1.58)
	Parekh [20], 2020		2016–17	0.69 (0.30–1.56)
COPD				
COPD, emphysema, chronic bronchitis	Antwi [21], 2022	BRFSS	2018	1.53 (1.08–2.16)
	Barrameda [22], 2021		2016	1.83 (1.59–2.10)
	Bircan [23], 2021		2016–18	1.44 (1.42–1.46)
	Osei et al. [24], 2020		2016–17	1.75 (1.14–2.69)
	Parekh [25], 2020		2016–17	1.37 (0.59–3.16)
	Wills [26], 2022		2020	1.44 (1.21–1.71)
	Xie [27], 2020a		2016–17	1.47 (0.92–2.36)
	Wills [28], 2019	Hawaii BRFSS	2016	2.58 (1.36–4.89)
Daily cough, sputum production, breathlessness	Giovanni [29], 2020	BRFSS	2017	1.36 (1.02–1.82)
COPD COPD, emphysema, CB	Cordova [30], 2022	PATH	W1-4	6.50 (3.25–12.99)
	Perez [31], 2019b		W1	1.43 (1.07–1.91)
COPD, CB, emphysema, asthma, some other respiratory condition	Strong [32], 2018		W1	1.39 (1.06–1.83)
Long-standing cough, sputum production, chronic productive cough, any wheeze, recurrent wheeze	Hedman [33], 2018	OLIN and WSAS	2016	1.46 (0.93–2.29)

*HF* Heart Failure

*MI* Myocardial Infarction

*COPD* chronic obstructive pulmonary disease

*PATH* Population Assessment of Tobacco and Health

*NHIS* National Health Interview Survey

*BRFSS* Behavioral Risk Factor Surveillance System

*OLIN* The Obstructive Lung Disease in Northern Sweden study

*WSAS* West Sweden Asthma Study

*W* PATH wave

*RR* relative risk

*OR* odds ratio

*CI* 95% confidence interval. Values include Bonferroni adjustments by Glantz et al

**Table 2:** Characteristics of Longitudinal Studies Used in Meta-Analysis of Cardiovascular Disease, Stroke and COPD Among E-cigarette Users Vs. Nonusers. Glantz et al. [3], Figure S2, Supplement

Glantz Disease Category Detailed study outcome(s)	Study First Author (Reference), year			Data source		Year/Wave(s)				RR/OR (CI)
Cardiovascular disease										
MI (from medical record)		Goldberg-Scott [15], 2023			Kaiser Permanente			2015–19	1.30 (0.66–2.55)	
MI or needed bypass surgery, HF, other heart condition, stroke		Berlowitz [16], 2022			PATH			W1-5	1.00 (0.64–1.57)	
MI or needed bypass surgery		Hirschtick [17], 2022							0.61 (0.08–4.39)	
MI, high blood pressure, high cholesterol, stroke, congestive HF, or some other heart condition		Qeadan [18], 2023							1.02 (0.90–1.15)	
Stroke										
Stroke		Hirschtick [17], 2022			PATH			W1-5	1.74 (0.55–5.47)	
Stroke (from medical record)		Goldberg-Scott [15], 2023			Kaiser Permanente			2015–19	1.65 (0.94–2.89)	
COPD										
COPD, CB, emphysema, asthma COPD, CB, emphysema COPD,	Bhatta [34], 2020		PATH				W1-3		1.44 (0.79–2.62)	
	Cook [35], 2023						W1-5		1.10 (0.74–1.64)	
	Paulin [36], 2022						W1-5		1.36 (0.48–3.85)	
	Xie [37], 2020b						W1-4		1.57 (1.08–2.29)	
Asthma, COPD, CB, emphysema, some other lung or respiratory condition	Qeadan [18], 2023						W1-5		1.11 (0.97–1.27)	
Influenza (from medical record)	Goldberg-Scott [15], 2023		Kaiser Permanente				2015–9		0.96 (0.71–1.30)	

*MI* Myocardial Infarction

*HF* Heart Failure

*COPD* chronic obstructive pulmonary disease

*CB* chronic bronchitis

*PATH* Population Assessment of Tobacco and Health

*W* PATH wave

*RR* relative risk

*OR* odds ratio

*CI* 95% confidence interval. Values include Bonferroni adjustments by Glantz et al

With respect to the CVD category, the most flagrant error was the inclusion in the meta-analysis of a study [3] of an irrelevant disease, erectile dysfunction (ED). Although ED may involve blood vessels and may be associated with smoking [38], it is not in any way associated with the other more serious and life-threatening or even fatal CVDs in this list, and its inclusion was not justified by Glantz et al. While most ORs for CVD ranged from 0.61 to 1.79 with only one reporting statistical significance (1.79, CI 1.20–2.67), the OR for ED is the largest in the CVD category (OR = 2.24, CI 1.50–3.34). As a result, it likely had a substantial impact by increasing the pooled OR for CVD among e-cigarette users versus nonusers (OR = 1.24, CI 1.05–1.46).

Glantz’s list of detailed outcomes in Figure S2 is inaccurate. For one study [9] they listed HF instead of congestive heart failure; in another [17] they listed MI instead of MI or needed bypass surgery. In four other studies [13, 14, 16, 18] they listed the detailed outcomes as coronary heart disease, MI, stroke, CVD. The latter acronym is confusing because it is the name of the category. Outcomes in two of the studies [13, 14] were coronary heart disease, MI and stroke, but the other two studies [16, 18] added several other conditions (Table 2).

Glantz et al. did not use one of the established frameworks for combining outcomes related to major adverse cardiovascular events (MACE) [39]. In contrast, the CVD category in Glantz et al. demonstrates considerable heterogeneity. It contained 4 studies with only myocardial infarction as the outcome [10–12, 15], and six other studies, five of which contained MI plus a diverse list of other diagnoses [13–15, 17, 18]. Two of these studies included “needed bypass surgery,” for which there is no ICD code [16, 17]. One study [18] included “high blood pressure,” “high cholesterol,” “or some other heart condition.” Three studies in the CVD category included “stroke,” [13, 14, 18] an outcome that Glantz et al. placed in a completely different category.

Glantz et al. also mixed outcomes in the COPD category. Many included COPD, emphysema and chronic bronchitis, which is defensible due to overlapping phenotypes [40], and if applied consistently. But Glantz et al. also inexplicably included studies with mixed outcomes under a descriptor “Respiratory symptoms.” There was no agreement in specific outcomes among four of the five studies [15, 18, 29, 32, 33]. Two added asthma and other lung or respiratory conditions to the previous trio [18, 32], while the outcomes from Giovanni [29] and Hedman [33], such as daily, long-standing or chronic productive cough, sputum production, breathlessness and wheezing, do not necessarily pertain to COPD. The outcome from Goldberg-Scott [15] was influenza, which is caused by a virus and not remotely tied to COPD with respect to this meta-analysis; it should not have been included in the study.

Table S2 of the Glantz et al. Appendix, which provides detailed information about the studies they used, contains numerous obvious errors. For example, Table S2 incorrectly lists COPD as the only outcome in at least 6 studies [21, 23, 25–28]. In fact, it should have been COPD, emphysema or chronic bronchitis, because BRFSS combined these three diseases in a single question, and the datasets don’t permit separating them.

In sum, Glantz et al.’s meta-analysis failed to provide the precise definition of diseases, and it inappropriately combined diverse, and sometimes unrelated, diseases. The authors also failed to justify why they included outcomes such as ED and influenza. The inclusion of these outcomes, especially ED, led to upward bias of the pooled OR and misinterpretation of a relationship between e-cigarette use and diseases.

### Cross-sectional studies

Another critical issue with Glantz et al.’s research design is that a majority of the ORs (76% or 94/124) were obtained from cross-sectional studies of the following datasets: the National Health Interview Surveys (NHIS), the Behavioral Risk Factor Surveillance System (BRFSS), the PATH study, and two studies from Swedish cross-sectional datasets (Table 1).

The main problem with using NHIS and BRFSS cross-sectional data to examine the relationship between e-cig use and diseases is that both sets of variables are collected simultaneously. There is no information in these datasets about the age at initiation of e-cig and cigarette use and the age that participants were diagnosed with diseases [41]. Because cardiovascular and respiratory diseases and stroke occur primarily in middle age and older adults, it is essential to know the presence/absence and duration of exposure to smoke and vapor in order to estimate accurate ORs. Cross-sectional studies from NHIS and BRFSS do not have this information [41], so they should not have been included in the meta-analysis.

Even though the PATH study does contain age when first started smoking or vaping and age of first disease diagnosis, the five studies selected by Glantz et al. in Table 1 [8, 9, 30–32] did not use this crucial information. Hence, they should not have been part of the meta-analysis.

### Longitudinal studies

Although the nine studies in Table 2 using PATH longitudinal follow-up data had the benefits of information about pre-existing diseases at enrollment, age at vaping and cigarette smoking initiation and duration, and changes in vaping/smoking status at follow-up waves, poor research design could undermine these advantages. For example, researchers should account for changes in vaping and smoking throughout follow-up waves, regardless of whether exclusive e-cigarette users at the baseline had never smoked (Plurphanswat et al., Submitted for publication). However, only the study by Berlowitz et al. [16] adjusted for time-varying current tobacco use, and their findings were not statistically significant.

Eight of 9 longitudinal studies reported no relationship between e-cigarette use and CVD, stroke and COPD. Our analysis of Xie et al. [37], the only study reporting a significant result, illustrates critical research design problems.

First, we established our study sample using Xie’s criteria, which were adults who were in Waves 1 to 4, had no respiratory conditions (COPD, emphysema, chronic bronchitis, or asthma) in Wave 1. We found that there were 2,827 current e-cig users at Wave 1; 161 of them reported that they had COPD, of whom 138 were current or former smokers. If this sample is restricted to never smokers of 100 + cigarettes (i.e. the standard definition of never smokers) (Table 3), the number of current e-cig users drops to 506. However, 285 of them were current cigarette triers (had not smoked 100 cigarettes but smoked every day or some days), 169 were former cigarette triers (had not smoked 100 cigarettes and did not smoke at the survey), and only 52 had never smoked, even one or two puffs. Table 3 lists these e-cig users’ smoking status and COPD status at Wave 1.

**Table 3:** Smoking status and COPD of 2827 current E-cigarette users at PATH Wave 1

Smoking status	Current E-cigarette	Had COPD (%)
Never smoked	506	23 (4.5)
Never even one puff	52	0 (0.0)
Current triers	285	20 (7.0)
Former triers	169	3 (1.8)
Current	1975	115 (5.8)
Former	346	23 (6.6)
Total	2827	161

*COPD* chronic obstructive pulmonary disease

*PATH* Population Assessment of Tobacco and Health

We then excluded e-cig users in Wave 1 who reported COPD (*n* = 161), and we followed the remaining 2,661 e-cig users in Waves 2–4. Among these participants, 35 cases of COPD were reported in Wave 2, 48 cases in Wave 3 and 61 cases in Wave 4. Table 4 shows the distribution of these cases according to e-cig and smoking status.

**Table 4:** E-cigarette and smoking status among wave 1 E-cigarette users without COPD at PATH Wave 1 Who reported COPD in Waves 2–4

E-cigarette status	Smoking status	COPD cases		
		Wave 2	Wave 3	Wave 4
Current	Current	15	19	19
Current	Former	5	4	5
**Current**	**Never**	**1**	**0**	**1**
Former	Current	8	14	27
Former	Former	0	6	5
Former	Never	0	0	1
Missing	Current	4	4	2
Missing	Former	2	1	1
Total		35	48	61

If the E-cigarette and smoking status categories are not in the table, the values are 0 values for all waves

*COPD* chronic obstructive pulmonary disease

*PATH* Population Assessment of Tobacco and Health

Although 69 COPD cases are seen in follow-up waves among current e-cig users, all but one are current or former smokers in the wave. Therefore, the results of Xie et al. [37] were almost entirely confounded by smoking.

## Discussion

Our investigation of the meta-analysis by Glantz et al. demonstrates that it is misleading and inaccurate, for three fundamental reasons: it inexplicably mixed disease outcomes; it used cross-sectional studies that are not adequate to credibly address associations, let alone casual relationships [42]; and it used poorly analyzed longitudinal studies.

To the casual reader, the meta-analysis may appear to be competently performed and tested, but this is deceptive [4, 5]. The deficits are only apparent to investigators thoroughly experienced with the data from the source studies. In addition to using inaccurate data, the meta-analysis has several statistical issues that appear to be addressed but are not.

The first author of the meta-analysis is fully knowledgeable of these critical problems, because he has used NHIS and PATH to co-author source studies. More importantly, he previously ignored established temporal information in PATH [43], resulting in retraction of his study on vaping and myocardial infarction in the *Journal of the American Heart Association* [44].

Glantz et al. recognized that they included multiple studies using the same dataset, and we agree that some form of correction is necessary. But they performed a Bonferroni correction. The only reference they list for this procedure is one that Glantz co-authored, but it provides no theoretical support for using Bonferonni in the meta-analysis context. Furthermore, Glantz et al. said they followed “advice for handling duplicate studies in meta-reviews,” but they did not use any of the techniques from the papers they cited.

Other analytic problems exist. Glantz et al. employed a meta regression claiming that there was no difference between the longitudinal and cross-sectional studies. Despite “controlling for” outcomes, their regression allowed for differences only in the intercept of the regression line for each one. This means that each outcome shared the same coefficients for the covariates in the model (i.e. the coefficient for cross-sectional/longitudinal took on the same value for every outcome). Thus, they did not sufficiently control for that variable when drawing conclusions about study characteristics (e.g. the comparability of cross-sectional and longitudinal studies). Furthermore, the coefficients and confidence intervals for any variable in a meta regression are interpreted with respect to every other included variable. Careful interpretation requires thoroughly examining diagnostics on the meta regression and correlation analysis between the included covariates, but these were not provided. Finally, our previous analysis of cross-sectional studies proves that they should never have been included in the meta analysis.

Statistical techniques can never rescue bad practice, but at the very least an attempt to include cross-sectional studies, even incorrectly, should place the burden of proof on showing that there is no difference between cross-sectional and longitudinal studies. This calls for an equivalence test [45], which was not performed. Instead, Glantz et al. interpreted “not statistically significant” as “accepting” the null hypothesis, mistakenly assuming a priori that cross-sectional and longitudinal studies qualitatively produce equivalent and comparable results.

There is another, expansive and serious shortcoming in both this meta-analysis and its source studies: the adverse health effects caused by cigarette smoking are observed in middle-age and older adults after decades of exposure [46–49]. Not only are almost all middle-age or older e-cigarette users long-term current or short-term former smokers, the adverse health effects of smoking at the population level were not documented for decades after smoking became popular. It is unrealistic for the adverse effects resulting from inhaling e-cigarette vapor, which is far less toxic than smoke [50], to be observed after only a few years of vapor exposure almost completely confounded by smoke exposure. The only reliable information will come after years of observation of e-cigarette users who started as youth or young adults, and never smoked.

In this study we documented several serious flaws in the meta-analysis by Glantz et al. [3], including heterogeneous outcomes in the CVD and COPD categories, the inappropriate inclusion of cross-sectional studies, and the use of results from a longitudinal study almost entirely confounded by smoking. In short, Glantz et al. failed to meet basic criteria for the quality of source studies [6, 7]; the results of their meta-analysis are invalid.
